# Supplementary material for: From sequence to enzyme mechanism using multi-label machine learning
Source: BMC Bioinformatics. 2014 May 19;15:150. doi: 10.1186/1471-2105-15-150 (PMC4229970; doi:10.1186/1471-2105-15-150)
Supplement: Additional file 2 — Java code of ml2db. Additional file ml2db_code.tar.gz contains the Java source code to run the multi-label machine learning experiments and save the results to database. The code’s Javadoc is included. [file 1471-2105-15-150-S2.zip › additional file 2/ml2db/utils/doc/uk/ac/standrews/utils/main/stats/UniformRandomUtils.html]

UniformRandomUtils


JavaScript is disabled on your browser.


- Overview
- Package
- Class
- Use
- Tree
- Deprecated
- Index
- Help

- Prev Class
- Next Class

- Frames
- No Frames

- All Classes

- Summary:
- Nested |
- Field |
- Constr |
- Method

- Detail:
- Field |
- Constr |
- Method


uk.ac.standrews.utils.main.stats

## Class UniformRandomUtils

- java.lang.Object
- - uk.ac.standrews.utils.main.stats.UniformRandomUtils

- ---

    

  ```
  public class UniformRandomUtils
  extends java.lang.Object
  ```

  Utilities for extracting with uniform random distribution

  Version:
  :   29 Jun 2008

  Author:
  :   Luna De Ferrari luna.deferrari-at-ed.ac.uk

- - ### Constructor Summary

    Constructors

    Constructor and Description

    `UniformRandomUtils()`
  - ### Method Summary

    Methods

    | Modifier and Type | Method and Description |
    | --- | --- |
    | `static java.lang.Double` | `extractDoubleBetween0And1()` Returns a random double between 0 and 1 |
    | `static int` | `extractInteger(int size)` Returns a random integer between 0 and size-1 |
    | `static java.util.Map.Entry` | `extractUniformRandomMapEntry(java.util.Map map)` Extracts a random entry (key-value association) from a map |
    | `static java.util.Map` | `getUniformRandomSubmap(java.util.Map map, double percentage)` Returns a submap of randomly picked map entries |
    | `static java.lang.Object` | `uniformPick(java.util.Vector collection)` |

    - ### Methods inherited from class java.lang.Object

      `equals, getClass, hashCode, notify, notifyAll, toString, wait, wait, wait`

- - ### Constructor Detail


    - #### UniformRandomUtils

      ```
      public UniformRandomUtils()
      ```
  - ### Method Detail


    - #### extractDoubleBetween0And1

      ```
      public static java.lang.Double extractDoubleBetween0And1()
      ```

      Returns a random double between 0 and 1

      Parameters:
      :   `size` - size-1 is the biggest integer that can be extracted

      Returns:
      :   a random integer between 0 and (size-1)


    - #### extractInteger

      ```
      public static int extractInteger(int size)
      ```

      Returns a random integer between 0 and size-1

      Parameters:
      :   `size` - size-1 must be a positive integer and is the biggest integer
          that can be extracted

      Returns:
      :   a random integer between 0 and size-1


    - #### extractUniformRandomMapEntry

      ```
      public static java.util.Map.Entry extractUniformRandomMapEntry(java.util.Map map)
      ```

      Extracts a random entry (key-value association) from a map

      Parameters:
      :   `map` - a map

      Returns:
      :   a random (uniform probability) entry from the map


    - #### getUniformRandomSubmap

      ```
      public static java.util.Map getUniformRandomSubmap(java.util.Map map,
                                         double percentage)
      ```

      Returns a submap of randomly picked map entries

      Parameters:
      :   `map` - the original map
      :   `percentage` - the percentage of the original map to be put in the submap

      Returns:
      :   the random submap


    - #### uniformPick

      ```
      public static java.lang.Object uniformPick(java.util.Vector collection)
      ```


- Overview
- Package
- Class
- Use
- Tree
- Deprecated
- Index
- Help

- Prev Class
- Next Class

- Frames
- No Frames

- All Classes

- Summary:
- Nested |
- Field |
- Constr |
- Method

- Detail:
- Field |
- Constr |
- Method
